# Exploring factors associated with high frequency emergency department use by children and young people: a retrospective cohort study

**DOI:** 10.1136/bmjpo-2025-003988

**Published:** 2026-07-15

**Authors:** Akshay Kumar, Rebecca M Simpson, Kerryn Husk, Graham D Johnson, Chris Burton

**Affiliations:** 1 Division of Applied Health Research Methods, University of Leeds, Leeds, UK; 2 ScHARR, The University of Sheffield, Sheffield, UK; 3 NIHR CLAHRC South West Peninsula (PenCLAHRC), University of Plymouth, Plymouth, UK; 4 Emergency Department, University Hospitals of Derby and Burton NHS Foundation Trust, Derby, UK; 5 School of Medicine, University of Nottingham, Nottingham, UK

**Keywords:** Child Health

## Abstract

**Objective:**

To explore drivers of high frequency emergency department (ED) use by children and young people, particularly among those attending EDs most often.

**Design:**

A population-based retrospective cohort study using routinely collected linked healthcare data.

**Setting:**

EDs in the Yorkshire and Humber region of the UK.

**Patients:**

288 545 children and young people aged 17 and under making at least one ED attendance in the year period 1 April 2016 to 31 March 2017.

**Main outcome measures:**

ED attendances made by individuals in the year period.

**Results:**

Of the 288 545 distinct children and young people making at least one attendance to an ED, 27 560 (9.6%) were defined as high frequency ED attenders (making 3+attendances in the year) and accounted for 105 063 (25%) ED attendances in the study period. When considering factors associated with ED attendance rates, younger age groups and greater levels of deprivation were found to be associated with higher rates of attendance. This association was amplified in those attending the ED most often. Ethnicity was found to be associated with ED attendance rates, with those reported as mixed and other ethnicities displaying greater rates of attendance than those reported as white and those reported as black ethnicity displaying lower attendance rates in comparison to those reported as white. The interaction between an individual’s deprivation and ethnic status generally displayed lower rates of attendance in ethnic minorities belonging to the most deprived category.

**Conclusions:**

When seeking to reduce frequent ED attendance by children and young people this study highlights the need for any approach to be multifaceted, nuanced to age or condition specifics and consider social determinants that drive ED use in more deprived areas.

**WHAT IS ALREADY KNOWN ON THIS TOPIC**

- High frequency emergency department (ED) users make a disproportionately large number of ED attendances, with the majority of previous research focusing on adult frequent attenders. Less is known about high frequency ED use by children and young people in the UK.

**WHAT THIS STUDY ADDS**

- Those defined as high frequency ED users were the youngest children and often the most socioeconomically deprived individuals. When specifically considering individuals responsible for the most ED attendances this association was amplified, however older individuals also displayed high rates of ED attendances.

**HOW THIS STUDY MIGHT AFFECT RESEARCH, PRACTICE OR POLICY**

- The findings of this study highlight the need for any approach to be multifaceted, nuanced to age or condition specifics and take into account social determinants that drive ED use in more deprived areas.

## Background

 Visits to hospital-based services such as emergency departments (EDs) have increased in recent years, with unscheduled attendances rising by over 15% in the last decade.^1^ Due to the potential consequences of an unsustainable rise in ED use on resources and patient care, there has been notable interest among researchers to explore high frequency ED use.^2 3^ While there is no universally accepted threshold for identifying high frequency ED attenders, studies generally define them as those who make three or more ED attendances within a specified time frame-typically a year.^4^ The reasons behind why individuals frequently attend EDs represent a complex interplay of medical, socioeconomic, psychological and healthcare system factors.^5^ When considering children and young people (C&YP) in particular, this is further complicated by parental/caregiver decision-making, which encompasses important considerations such as the perceived urgency of the child’s healthcare need, in addition to other factors such as perceived ED advantages (including faster service, superior ED resources and efficiency), convenience and lack of access to primary care services.^6 7^ Medical factors also play an important role in high frequency ED use, as one study found that frequently attending C&YP were more likely to be younger children suffering from one or more chronic conditions.^4^ A published systematic review outlining characteristics of frequently attending C&YP found they were also more likely to be high frequency users of primary care services, with deprivation being associated with high frequency ED use.^5 8 9^ However, this was generally examined through the use of medical insurance with only one study exploring this in the UK.^10^ The UK-based study suggested that further information regarding frequent ED use by C&YP could be provided by analysing smaller regions of the UK to explore more granular data, such as ED outcomes.

When considering high frequency ED attendance in adults, previous studies have outlined the heavy tailed nature of the ED attendance distribution.^11^ This suggests that although a large proportion of individuals make a small number of attendances, a notable proportion of individuals attend EDs very frequently. In the design of this study, this hypothesis was tested and a similar heavy tail distribution was found for C&YP. Hence, this study aimed to specifically explore factors associated with ED use for C&YP belonging to the upper tail of the ED attendance distribution and therefore attend EDs the most often.

The objective of this study is to:

Examine factors associated with rates of ED attendance in C&YP, with additional emphasis placed on those attending ED the most often.

The authors would like to acknowledge that parts of the content presented in this manuscript are drawn from chapter 8 of Dr Akshay Kumar’s doctoral thesis titled ‘Examining patterns of urgent and emergency care service use by children and young people. PhD thesis, (University of Sheffield, 2023)’.^12^ This doctoral thesis also contributed to the publication of two other manuscripts exploring ED use by C&YP.^3 13^

## Methods

### Study design

A population-based retrospective cohort study using routinely collected linked healthcare data.

### Data used


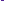

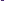
This study used data collected from the ‘Connected Health Cities: Data linkage of urgent care data’ study (known as the ‘CUREd research database’).^14^ The CUREd research database holds data from National Health Service (NHS) 111 calls, emergency ambulance incidents, ED attendances and emergency admissions to hospitals in the Yorkshire and Humber region of the UK. Each entry has an anonymised common patient identifier code to facilitate linkages across the datasets.^15^ This study used data from the ED dataset (420 051 attendances) for C&YP aged 17 and under, in the year period 1 April 2016 to 31 March 2017. The ED dataset consisted of patient records (items mandated by the national commissioning dataset) for attendances made to the 13 participating hospital trusts’ EDs, urgent care centres and walk-in-centres.

### Patient and public involvement

Patients were not directly involved in the planning or execution of this research which analysed routinely collected healthcare data. However, patient and public involvement plays a pivotal part in the conceptualisation and collection of the CUREd research database and uses the assistance of a Data Release Committee (DRC) which acts as an oversight panel for the CUREd platform, including patient and public representation, healthcare stakeholders and information governance specialists. The DRC reviewed this study which was designed to be a secondary data analysis based on a noted literature gap.

### Data management

#### Data extraction

The data extraction, cleaning, linking and statistical analyses were performed using R (V.4.2.1). From the ED attendance dataset, the following items were extracted: Anonymised patient identifier, encrypted attendance record identifier, sex of the patient, ethnicity of the patient, age at each attendance, attendance disposal, arrival date/time, conclusion time, low acuity attendance indicator and a patient’s incident Index of Multiple Deprivation (IMD). The distribution of patients by IMD quintiles in the data was not even; this reflects both the demography of Yorkshire and Humber generally—with more people living in deprived areas than the English average and greater ED use by people of lower socio-demographic status.

#### Data preparation

An individual’s age was taken at their first attendance in the study period and categorised into the following groups to align with previously published research: <1, 1–4, 5–9, 10–14 and 15–17.^8^ An ED attendance count variable was derived by aggregating the number of ED attendances an individual made to any ED in the Yorkshire and Humber region within the year study period. When examining the <1 age group, the calculated outcomes (such as incidence rate ratios (IRRs)) may tend to underestimate the actual values. This is because these individuals may not have had the opportunity to visit the ED, due to their later birth within the study period. Descriptive analyses were conducted on both attendance-level data and individual-level data, whereas regression models were fit using individual level data only. Regression analyses were conducted using the complete-case sample and thus individuals with missing data were omitted from this analysis.

### Definitions

#### High frequency ED attender

A high frequency ED attender was defined as an individual making three or more ED attendances in a year period, otherwise they were defined as an occasional attender.

#### Attendance acuity

A low acuity ED attendance field was defined as an attendance made to a type 1 ED meeting the following three conditions:^16^

- Attendance produced at least one of the following investigation codes: none, urinalysis, pregnancy test or dental investigation.
- Attendance produced at least one of the following treatment codes: prescription(s), guidance/advice only, recording vital signs, dental treatment or no investigation.

- Attendance produced at least one of the following disposals: discharged (following treatment to be provided by general practitioner (GP)/no follow-up treatment required) or left department before being treated.

A high acuity ED attendance was defined as an attendance that resulted in the patient being

admitted to hospital, and an intermediate acuity ED attendance was defined as all attendances not falling into the low or high acuity attendance category. When considering low acuity ED attendances in this study, it is possible that the patient was correctly seen in the ED (eg, some patients i may have attended the ED at the explicit instruction of a healthcare professional, even though they subsequently met the definition of a low acuity attendance).

### Statistical analysis

#### Factors associated with ED attendance rates

We found that the yearly ED attendances made by C&YP likely followed a heavy tail distribution (such as a discrete power law). We therefore used a zero truncated Poisson regression model to determine factors associated with ED use for the majority of the data, in combination with a quantile regression model to determine factors associated with ED use for those attending most often.^17^ A logistic regression model was also fit to determine the odds of an individual being a frequent attender using the binary definition of frequent attendance (3+attendances in the year). The zero truncated Poisson model was chosen after comparing the fit of a number of count-generalised linear regression models. Quantile regression (for count data) was used to assess the association between patient factors and ED attendance rates specifically for individuals belonging to the upper tail of the attendance distribution. As we were interested in exploring individuals attending ED the most often, the 90th, 92nd, 94th, 96th, 98th, 99th, 99.5th and 99.9th quantiles were chosen in addition to the median for reference. The patient’s sex, age, ethnicity and deprivation status were independent variables considered in all statistical analyses taken at their first ED attendance. An interaction term between ethnicity and deprivation was also evaluated in the Poisson and logistic regression analyses. The resulting IRRs, ORs and corresponding 95% CIs were reported.

## Results

### Patient and attendance characteristics

In the period 1 April 2016 to 31 March 2017, 420 051 ED attendances were made to hospitals in the Yorkshire and Humber region of the UK by 288 545 distinct C&YP. Of these individuals, 27 560 (9.6%) were defined as high frequency ED attenders (making 3+attendances in the year) and were responsible for 105 063 (25%) ED attendances in the study period. High frequency attenders were more likely to be younger children, with 4732 (17.2%) being under the age of 1 at the time of their first attendance, in comparison to 24 307 (9.3%) occasional ED attenders belonging to the same age group. When considering the deprivation status of an individual, high frequency attenders were more likely to belong to the more deprived groups than occasional attenders and less likely to belong to the least deprived group (table 1). Attendances made by high frequency attenders were more likely to be defined as high acuity (17 917, 17.1%) in comparison with attendances made by occasional attenders (36 951, 11.7%). High frequency attenders were slightly more likely to have been referred to an ED by a GP and arrive by ambulance in comparison with occasional attenders (table 2).

**Table 1:** Patient characteristics for occasional and high frequency users

Characteristics	Occasional ED users (<3) N=260 985 (90.4%)			High frequency ED users (≥3) N=27 560 (9.6%)		
	**n**	**%**	**95%** CI	**n**	**%**	**95%** CI
Sex						
Male	143 121	54.8	54.6 to 55.0	15 332	55.6	55.2 to 56.0
Female	117 853	45.2	45.0 to 45.4	12 228	44.4	44.0 to 44.8
Missing	11	0.004	-	-	-	-
Age group						
<1	24 307	9.3	9.2 to 9.4	4732	17.2	16.8 to 17.6
1–4	69 144	26.5	26.4 to 26.6	8009	29.0	28.7 to 29.3
5–9	57 996	22.2	22.1 to 22.3	4140	15.0	14.8 to 15.3
10–14	58 457	22.4	22.3 to 22.5	5987	21.7	21.3 to 22.1
15–17	33 329	12.8	12.7 to 12.9	3460	12.6	12.3 to 12.9
Missing	17 753	6.8	–	1232	4.5	–
Ethnicity						
White	191 825	73.5	73.4 to 73.6	20 404	74	73.8 to 74.2
Asian	29 668	11.4	11.3 to 11.5	3556	12.9	12.7 to 13.1
Black	4020	1.5	1.5 to 1.5	442	1.6	1.5 to 1.7
Mixed ethnicity	7531	2.9	2.9 to 3.0	997	3.6	3.5 to 3.7
Other ethnicities	7811	3.0	2.9 to 3.0	1009	3.7	3.6 to 3.8
Missing	20 130	7.0	–	1152	4.2	–
IMD status						
1 (most deprived)	108 737	41.7	41.6 to 41.8	13 415	48.7	48.4 to 49.0
2	46 969	18.0	17.9 to 18.1	4996	18.1	17.8 to 18.4
3	37 162	14.2	14.1 to 14.3	3626	13.3	13 to 13.6
4	37 472	14.4	14.3 to 14.5	3200	11.6	11.4 to 11.9
5 (least deprived)	29 148	11.2	11.1 to 11.3	2282	8.3	8.1 to 8.5
Missing	1497	0.6	–	41	0.1	–

ED, emergency department; IMD, Index of Multiple Deprivation.

**Table 2:** ED attendance characteristics for occasional and high frequency users

Characteristics	Attendances made by occasional ED users (<3) N=314 988			Attendances made by high frequency ED users (≥3) N=105 063		
	**n**	**%**	**95%** CI	**n**	**%**	**95%** CI
Acuity						
Low acuity	59 709	19.0	18.5 to 19.4	19 062	18.1	18.0 to 18.2
Intermediate acuity	173 509	55.1	54.7 to 55.4	49 083	46.7	46.6 to 46.8
High acuity	36 951	11.7	11.7 to 11.8	17 917	17.1	17.0 to 17.2
Missing	44 819	14.2	-	19 001	18.1	-
Referral						
GP	38 941	12.4	12.3 to 12.5	14 256	13.5	13.4 to 13.6
Self	227 124	72.1	71.9 to 72.2	76 031	72.4	72.3 to 72.5
Other/missing	48 923	15.5	15.4 to 15.6	14 776	14.1	14.0 to 14.2
Arrival mode						
Ambulance	34 897	11.1	11.0 to 11.2	14 367	13.7	13.6, 13.8
Other	272 592	86.5	86.4 to 86.6	88 630	84.4	84.3 to 84.5
Missing	7499	2.4	–	2066	1.9	–

ED, emergency department; GP, general practitioner.

### Factors associated with high frequency ED use

Children under the age of 1 displayed higher rates of attendance in comparison to individuals aged 15–17 (IRR=1.40, 95% CI 1.38 to 1.43) and children aged 5–9 displayed lower rates of attendance in comparison to those aged 15–17 (IRR=0.73, 95% CI 0.72 to 0.74). Those belonging to the most deprived category were found to have higher rates of attendance in comparison to those belonging to the least deprived groups (IRR=1.30, 95% CI 1.27 to 1.33). An association was found between those reported as mixed ethnicity (IRR=1.07, 95% CI 1.04 to 1.10) and other ethnicities (IRR=1.10, 95% CI 1.07 to 1.13), with these individuals displaying higher rates of attendance than those reported as white ethnicity. Additionally, those reported as black ethnicity were associated with lower rates of ED attendances in comparison to those reported as white ethnicity (IRR=0.95, 95% CI 0.91 to 0.99). There was no statistically significant association found between those reported as Asian ethnicity (IRR=1.01, 95% CI 0.99 to 1.03) and rates of ED attendance in comparison to white ethnicity (table 3). The interaction between an individual’s deprivation and ethnic status generally displayed lower rates of attendance for ethnic minorities belonging to the most deprived category. However, this was not seen in those reported as black ethnicity (online supplemental material). There was a small but statistically significant association between the sex of an individual and the rates of attendance with females having lower rates of attendance than males (IRR=0.98, 95% CI 0.97 to 0.99). The odds of being a frequent attender found when computing the logistic regression model mirrored the rate ratios reported above (table 3).

**Table 3:** Logistic regression and zero-truncated Poisson regression model incidence rate ratios

	Logistic regression		ZT Poisson regression	
Predictors	OR	95% CI	IRR	95% CI
Sex				
Male (REF)				
Female	0.96	0.94 to 0.99	0.98	0.97 to 0.99
Age				
<1	2.70	2.58 to 2.82	1.92	1.88 to 1.95
1–4	1.61	1.54 to 1.67	1.36	1.34 to 1.38
5–9 (REF)	1.00	1.00 to 1.00	1.00	1.00 to 1.00
10–14	1.48	1.42 to 1.54	1.31	1.29 to 1.34
15–17	1.52	1.45 to 1.60	1.38	1.35 to 1.40
Deprivation				
5 (least deprived, REF)				
4	1.04	0.98 to 1.10	1.04	1.02 to 1.07
3	1.20	1.13 to 1.27	1.12	1.09 to 1.15
2	1.28	1.21 to 1.35	1.19	1.17 to 1.22
1 (most deprived)	1.46	1.39 to 1.54	1.30	1.27 to 1.33
Ethnicity group				
White (REF)				
Asian	1.02	0.98 to 1.06	1.01	1.00 to 1.03
Black	0.91	0.82 to 1.01	0.95	0.91 to 0.99
Mixed	1.11	1.03 to 1.19	1.07	1.04 to 1.10
Other	1.14	1.06 to 1.22	1.10	1.07 to 1.13
N=252 728				

REF, reference; ZT, zero truncated.

### Factors associated with ED attendance rates in those attending most often

**Figure 1:**
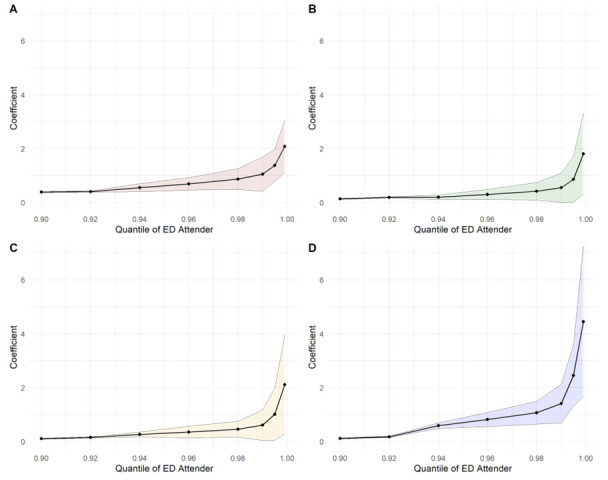
Quantile regression model coefficients displaying the association between age and ED attendance across higher quantiles of ED attenders, A=<1, B=1–4, C=10–14, D=15–17 (Ref=5–9). ED, emergency department.

**Figure 2:**
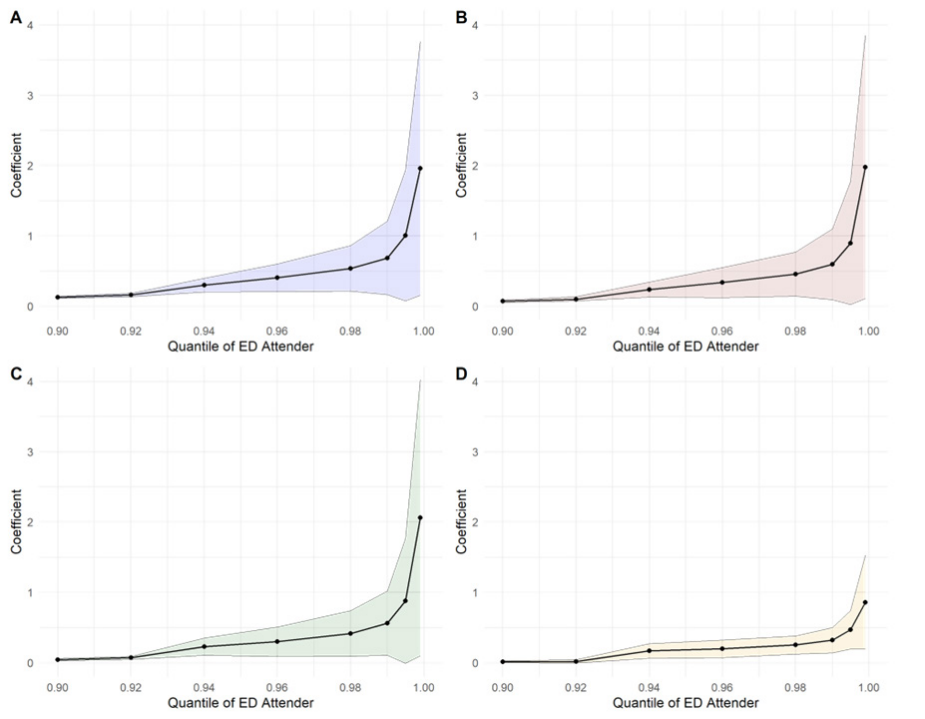
Quantile regression model coefficients displaying the association between deprivation and ED attendance across higher quantiles of ED attenders, A=1 most deprived, B=2, C=3, D=4 (Ref=5-least deprived). ED, emergency department.

Both those under the age of one and those aged 15–17 were more likely to be associated with higher rates of ED attendances specifically for those attending most often (figure 1). The association between deprivation and attendance rates was amplified in those attending most often (figure 2), however the association between an individual’s ethnicity and ED attendance diminished when considering these frequently attending individuals (online supplemental material).

## Discussion

### Summary of principal findings

A relatively small proportion of C&YP were defined as high frequency ED attenders (27 560, 9.6%) but accounted for a relatively large proportion of ED attendances (105 063, 25%).

High frequency ED attenders were more likely to be admitted to hospital after attending an ED than occasional users. When considering factors associated with ED attendance rates for C&YP, younger age groups and greater levels of deprivation were found to be associated with higher rates of attendance. This association was amplified for those attending ED most often. Ethnicity was found to be associated with ED attendance rates, with mixed and other ethnicities displaying greater rates of attendance than white individuals and black ethnicities displaying lower attendance rates in comparison to white individuals. The interaction between an individual’s deprivation and ethnic status largely displayed lower rates of attendance for ethnic minorities belonging to the most deprived category. With the exception of those belonging to the other ethnicities category, the association between ethnicity and rates of attendance diminished when considering those attending ED most often.

### Strengths and limitations

This study is the first known study to use a combination of a modified Poisson regression model and a quantile regression model when considering ED attendance. This is beneficial when analysing factors associated with ED attendance rates as it removes the need to categorise individuals into high frequency attenders based on an arbitrary threshold. The quantile regression conducted in this analysis provides a more dynamic picture of high frequency attendance by exploring factors associated with ED use for those attending most often. However, as there was a small number of individuals making a large number of attendances, sample sizes fell when considering higher quantiles of attenders. Hence, the CIs of quantile regression model outputs generally increased when considering those attending EDs the most often. Due to the lack of linkable primary care data, high frequency use of primary care services could not be explored as a factor associated with high rates of ED attendance in C&YP. Reasons for ED attendance were not included in this study due to a large proportion of missing diagnosis data and inconsistencies in the diagnosis classification system used across hospitals. As the data used in this study is over 8 years old, it may not reflect the recent behavioural and ED resource management shifts as a consequence of the COVID-19 pandemic. However, this study can be used as a pre-COVID-19 comparison with more up-to-date analyses.

### Comparison with other studies

Previous studies have found that younger age was associated with high frequency ED use in C&YP and this study adds support to these findings.^4^ This study further suggests that older age groups were equally likely to have high rates of attendance in comparison to other age groups when considering those who attend ED the most. One nationwide (England) study suggests ethnicity was a predictor of high frequency attendance for C&YP.^10^ The study similarly found that the odds of being a high frequency attender was lower for people reporting black ethnicities and greater for those reporting mixed ethnicities in comparison to those identifying as white. However, the nationwide study found the odds of being a high frequency attender higher in those reporting Asian ethnicities and lower in reported other ethnicities in comparison to individuals identifying as white. This implies there is likely to be variation between ethnicity and rates of ED attendance in different regions of the UK.

### Implication for practice and further research

Unlike adult high frequency ED use, which is often driven by chronic disease or substance misuse, paediatric ED use is often mediated by caregiver health literacy and risk perception. The finding that younger age groups and those from deprived areas attend more frequently suggests a ‘safety net’ reliance where the ED compensates for limited access to primary care or caregiver anxiety regarding acute illness in infants. To move toward proactive care, these data should inform risk stratification models that identify ‘rising risk’ families triggering early intervention, potentially via health visitors or community paediatric hubs before a pattern of high-frequency use is established.

This study has provided evidence to suggest there is a differential impact of age, ethnicity and deprivation on attendance rates across all ED users; however, high frequency user cohorts often have specific characteristics. Given the increase in repeated mental health presentations for older C&YP (with impacts of the COVID-19 pandemic exacerbating this problem), further research could explore this cohort in detail.^18^ Although there may be some evidence to suggest that a proportion of high frequency ED attenders suffer from chronic conditions, the association between increased deprivation and higher rates of ED attendance highlights the importance of also considering social determinants of health, which may suggest that healthcare providers could consider more preventive measures taking a more holistic approach that incorporates broader drivers of health and health inequalities. This study suggests that ED attendance is not consistent across ethnic groups, which highlights the need for further work to understand the complex intersectional mechanisms that drive use in areas of ethnic diversity. For example, future research could explore subdemographic factors such as the immigration status of ethnic minorities when considering ED use by C&YP.

## Conclusion

The findings of this study have highlighted the need for a multifaceted approach which targets age/condition specific interventions for parents and caregivers, and the social determinants that contribute to higher ED use in more deprived areas.

## Supplementary material

10.1136/bmjpo-2025-003988 online supplemental file 1

## Data Availability

Data may be obtained from a third party and are not publicly available.
