# Supplementary material for: Exploring factors associated with high frequency emergency department use by children and young people: a retrospective cohort study
Source: BMJ Paediatr Open. 2026 Jul 15;10(1):e003988. doi: 10.1136/bmjpo-2025-003988 (PMC13374425; doi:10.1136/bmjpo-2025-003988)
Supplement: online supplemental file 1 [file bmjpo-10-1-s001.docx]

Supplementary Material

*Table 1-Logistic and Zero Truncated (ZT) Poisson Regression with Interaction Term (Ethnicity & Deprivation) modelled using the binary high frequency attender (3+ attendances) and attendance count outcome variable respectively.*

|  | Logistic Regression | | ZT Poisson Regression | |
| --- | --- | --- | --- | --- |
| Predictors | OR | 95% CI | IRR | 95% CI |
|  |  |  |  |  |
| Sex |  |  |  |  |
| Male (REF) | 1.00 | 1.00-1.00 | 1.00 | 1.00-1.00 |
| Female | 0.98 | 0.97-0.99 | 0.98 | 0.97-0.99 |
|  |  |  |  |  |
| Age |  |  |  |  |
| <1 | 2.61 | 2.58–2.82 | 1.91 | 1.88-1.95 |
| 1-4 | 1.61 | 1.54–1.67 | 1.36 | 1.34-1.38 |
| 5-9 (REF) | 1.00 | 1.00-1.00 | 1.00 | 1.00-1.00 |
| 10-14 | 1.48 | 1.42–1.54 | 1.31 | 1.29-1.34 |
| 15-17 | 1.52 | 1.45–1.60 | 1.38 | 1.35-1.40 |
|  |  |  |  |  |
| Deprivation |  |  |  |  |
| 5 (Least Deprived, REF) | 1.00 | 1.00-1.00 | 1.00 | 1.00-1.00 |
| 4 | 1.06 | 1.02-1.07 | 1.05 | 1.02-1.07 |
| 3 | 1.19 | 1.09-1.15 | 1.13 | 1.09-1.15 |
| 2 | 1.29 | 1.17-1.23 | 1.20 | 1.17-1.23 |
| 1 (Most Deprived) | 1.51 | 1.43–1.59 | 1.33 | 1.27-1.33 |
|  |  |  |  |  |
| Ethnicity Group |  |  |  |  |
| White (REF) | 1.00 | 1.00-1.00 | 1.00 | 1.00-1.00 |
| Asian | 1.24 | 0.97-1.56 | 1.20 | 1.09-1.36 |
| Black | 0.95 | 0.91-0.99 | 0.91 | 0.67-1.24 |
| Mixed | 1.30 | 0.97-1.70 | 1.22 | 1.09-1.36 |
| Other | 1.36 | 0.99-1.82 | 1.27 | 1.12-1.43 |
|  |  |  |  |  |
| Ethnicity/Deprivation  Interaction  (REF: Deprivation 5-Least Deprived & White) |  |  |  |  |
| **Asian Ethnicity** |  |  |  |  |
| Deprivation [4] & Asian | 0.88 | 0.65 – 1.21 | 0.93 | 0.82 – 1.05 |
| Deprivation [3] & Asian | 1.04 | 0.79 – 1.38 | 0.95 | 0.85 – 1.06 |
| Deprivation [2] & Asian | 0.86 | 0.67 – 1.12 | 0.87 | 0.78 – 0.97 |
| Deprivation [1] & Asian | 0.79 | 0.62 – 1.02 | 0.82 | 0.74 – 0.90 |
| **Black Ethnicity** |  |  |  |  |
| Deprivation [4] & Black | 1.20 | 0.48 – 3.16 | 1.40 | 0.96 – 2.03 |
| Deprivation [3] & Black | 1.03 | 0.46 – 2.51 | 1.01 | 0.70 – 1.44 |
| Deprivation [2] & Black | 0.92 | 0.44 – 2.15 | 0.99 | 0.71 – 1.37 |
| Deprivation [1] & Black | 0.94 | 0.48 – 2.13 | 1.03 | 0.76 – 1.41 |
| **Mixed Ethnicity** |  |  |  |  |
| Deprivation [4] & Mixed | 0.80 | 0.55 – 1.16 | 0.82 | 0.71 – 0.96 |
| Deprivation [3] & Mixed | 0.89 | 0.63 – 1.27 | 0.86 | 0.75 – 1.00 |
| Deprivation [2] & Mixed | 0.89 | 0.65 – 1.24 | 0.93 | 0.81 – 1.06 |
| Deprivation [1] & Mixed | 0.83 | 0.62 – 1.12 | 0.86 | 0.76 – 0.97 |
| **Other Ethnicities** |  |  |  |  |
| Deprivation [4] & Other Ethnicities | 0.60 | 0.38 – 0.95 | 0.80 | 0.67 – 0.95 |
| Deprivation [3] & Other Ethnicities | 0.97 | 0.67 – 1.44 | 0.91 | 0.78 – 1.07 |
| Deprivation [2] & Other Ethnicities | 0.86 | 0.61 – 1.24 | 0.91 | 0.79 – 1.05 |
| Deprivation [1] & Other Ethnicities | 0.82 | 0.61 – 1.14 | 0.85 | 0.75 – 0.96 |
| N=252,728 | | | | |

Table 2-Output of the quantile regression for counts analysis across a range of quantiles

| Coefficient | Estimated Coefficients & Corresponding 95% CI (Quantiles) | | | | | | | | | | | | | | | | | | |
| --- | --- | --- | --- | --- | --- | --- | --- | --- | --- | --- | --- | --- | --- | --- | --- | --- | --- | --- | --- |
|  | Q(0.50) | CI | Q(0.90) | CI | Q(0.92) | 95% CI | Q(0.94) | 95% CI | Q(0.96) | 95% CI | Q(0.98) | 95% CI | Q(0.99) | 95% CI | Q(0.995) | 95% CI | Q(0.999) | 95% CI |  |
| **(Intercept)** | 0.119 | (0.114, 0.124) | 0.568 | (0.555, 0.582) | 0.603 | (0.583, 0.624) | 0.620 | (0.597, 0.643) | 0.773 | (0.732, 0.814) | 1.046 | (0.978, 1.114) | 1.232 | (1.190, 1.273) | 1.543 | (1.447, 1.639) | 2.159 | (1.951, 2.368) |  |
|  |  |  |  |  |  |  |  |  |  |  |  |  |  |  |  |  |  |  |  |
| **Sex (Ref=Male)** |  |  |  |  |  |  |  |  |  |  |  |  |  |  |  |  |  |  |  |
| Female | -0.012 | (-0.015, -0.009) | -0.012 | (-0.021, -0.003) | -0.010 | (-0.022, 0.001) | -0.009 | (-0.024, 0.006) | 0.000 | (-0.027, 0.027) | 0.028 | (-0.054, 0.109) | 0.055 | (-0.016, 0.125) | 0.090 | (-0.077, 0.258) | 0.163 | (-0.196, 0.522) |  |
|  |  |  |  |  |  |  |  |  |  |  |  |  |  |  |  |  |  |  |  |
| **Age (Ref=5-9)** |  |  |  |  |  |  |  |  |  |  |  |  |  |  |  |  |  |  |  |
| <1 | 0.133 | (0.126, 0.139) | 0.389 | (0.375, 0.403) | 0.408 | (0.391, 0.426) | 0.556 | (0.410, 0.702) | 0.693 | (0.463, 0.923) | 0.871 | (0.485, 1.258) | 1.057 | (0.424, 1.690) | 1.381 | (0.790, 1.971) | 2.086 | (1.118, 3.053) |  |
| 1-4 | 0.055 | (0.051, 0.058) | 0.142 | (0.129, 0.154) | 0.190 | (0.175, 0.207) | 0.196 | (0.109, 0.283) | 0.300 | (0.115, 0.486) | 0.419 | (0.093, 0.745) | 0.553 | (0.010, 1.097) | 0.867 | (0.012, 1.723) | 1.812 | (0.324, 3.299) |  |
| 10-14 | 0.043 | (0.039, 0.047) | 0.111 | (0.099, 0.123) | 0.155 | (0.136, 0.173) | 0.267 | (0.177, 0.357) | 0.352 | (0.134, 0.569) | 0.461 | (0.167, 0.756) | 0.611 | (0.041, 1.182) | 1.014 | (0.051, 1.977) | 2.115 | (0.279, 3.951) |  |
| 15-17 | 0.037 | (0.032, 0.041) | 0.119 | (0.104, 0.135) | 0.174 | (0.152, 0.197) | 0.592 | (0.490, 0.694) | 0.821 | (0.564, 1.078) | 1.070 | (0.650, 1.490) | 1.412 | (0.695, 2.129) | 2.451 | (1.323, 3.579) | 4.451 | (1.661, 7.241) |  |
|  |  |  |  |  |  |  |  |  |  |  |  |  |  |  |  |  |  |  |  |
| **Deprivation (Ref=5-Least Deprived)** |  |  |  |  |  |  |  |  |  |  |  |  |  |  |  |  |  |  |  |
| 4 | 0.007 | (0.001, 0.013) | 0.012 | (-0.002, 0.025) | 0.019 | (-0.006, 0.043) | 0.167 | (0.062, 0.272) | 0.197 | (0.072, 0.321) | 0.252 | (0.124, 0.380) | 0.320 | (0.141, 0.499) | 0.467 | (0.195, 0.740) | 0.859 | (0.193, 1.525) |  |
| 3 | 0.018 | (0.012, 0.024) | 0.044 | (0.029, 0.058) | 0.071 | (0.047, 0.088) | 0.229 | (0.107, 0.351) | 0.298 | (0.089, 0.506) | 0.415 | (0.093, 0.737) | 0.562 | (0.106, 1.018) | 0.878 | (-0.010, 1.765) | 2.059 | (0.098, 4.020) |  |
| 2 | 0.030 | (0.025, 0.036) | 0.070 | (0.053, 0.086) | 0.099 | (0.075, 0.134) | 0.236 | (0.129, 0.342) | 0.336 | (0.121, 0.550) | 0.457 | (0.143, 0.770) | 0.594 | (0.093, 1.096) | 0.897 | (0.025, 1.770) | 1.978 | (0.111, 3.845) |  |
| 1 (Most Deprived) | 0.046 | (0.041, 0.051) | 0.127 | (0.113, 0.141) | 0.159 | (0.136, 0.182) | 0.299 | (0.201, 0.398) | 0.404 | (0.209, 0.599) | 0.538 | (0.215, 0.862) | 0.686 | (0.163, 1.209) | 1.005 | (0.074, 1.936) | 1.958 | (0.158, 3.758) |  |
|  |  |  |  |  |  |  |  |  |  |  |  |  |  |  |  |  |  |  |  |
| **Ethnicity (Ref=White)** |  |  |  |  |  |  |  |  |  |  |  |  |  |  |  |  |  |  |  |
| Asian | 0.001 | (-0.004, 0.006) | 0.011 | (-0.004, 0.026) | 0.015 | (-0.003, 0.032) | 0.043 | (-0.046, 0.133) | 0.057 | (-0.065, 0.178) | 0.086 | (-0.094, 0.266) | 0.114 | (-0.119, 0.347) | 0.160 | (-0.159, 0.480) | 0.290 | (-0.207, 0.786) |  |
| Black | -0.012 | (-0.024, -0.001) | -0.035 | (-0.071, 0.002) | -0.039 | (-0.094, 0.012) | 0.050 | (-0.185, 0.171) | 0.088 | (-0.263, 0.439) | 0.134 | (-0.338, 0.607) | 0.184 | (-0.402, 0.769) | 0.282 | (-0.566, 1.130) | 0.567 | (-0.951, 2.086) |  |
| Mixed | 0.013 | (0.004, 0.022) | 0.049 | (0.019, 0.080) | 0.064 | (0.031, 0.097) | -0.012 | (-0.217, 0.193) | -0.013 | (-0.300, 0.273) | 0.012 | (-0.316, 0.340) | 0.040 | (-0.411, 0.491) | 0.101 | (-0.566, 0.768) | 0.283 | (-1.021, 1.588) |  |
| Other Ethnicities | 0.016 | (0.007, 0.025) | 0.044 | (0.017, 0.087) | 0.054 | (0.022, 0.089) | 0.156 | (-0.057, 0.369) | 0.199 | (-0.085, 0.483) | 0.251 | (-0.134, 0.636) | 0.313 | (-0.197, 0.823) | 0.453 | (-0.278, 1.183) | 0.797 | (-0.389, 1.983) |  |
