# Supplementary material for: Exploring factors associated with high frequency emergency department use by children and young people: a retrospective cohort study
Source: BMJ Paediatr Open. 2026 Jul 15;10(1):e003988. doi: 10.1136/bmjpo-2025-003988 (PMC13374425; doi:10.1136/bmjpo-2025-003988)
Supplement: Supplementary file 2 [file bmjpo-2025-003988.reviewer_comments.pdf]

PEER REVIEW HISTORY

BMJ Paediatrics Open publishes all reviews undertaken for accepted manuscripts. Reviewers are asked to complete a checklist review form and are provided with free text boxes to elaborate on their assessment. These free text comments are reproduced below.

ARTICLE DETAILS

**TITLE (PROVISIONAL)**	Exploring Factors Associated with High Frequency Emergency Department Use by Children & Young People: A Retrospective Cohort Study
**AUTHORS**	Kumar, Akshay<br>Simpson, Rebecca<br>Husk, Kerryn<br>Johnson, Graham<br>Burton, Chris

VERSION 1 – REVIEW

**REVIEWER NAME**	Reviewer 1
**REVIEWER AFFILIATION**	
**REVIEWER CONFLICT OF INTEREST**	No Conflict of Interest
**AI DISCLOSURE**	No, I have not used AI
**DATE REVIEW RETURNED**	12-Aug-2025

**GENERAL COMMENTS**	Comments not published pending reviewer's consent

**REVIEWER NAME**	Reviewer 2
**REVIEWER AFFILIATION**	
**REVIEWER CONFLICT OF INTEREST**	No Conflict of Interest
**AI DISCLOSURE**	No, I have not used AI
**DATE REVIEW RETURNED**	12-Aug-2025

**GENERAL COMMENTS**	Comments not published pending reviewer's consent

VERSION 2 – REVIEW

**REVIEWER NAME**	Rungan, Santuri
**REVIEWER AFFILIATION**	Croydon Community Mental Health Service, Community Paediatrics
**REVIEWER CONFLICT OF INTEREST**	No Conflict of Interest
**AI DISCLOSURE**	Chat GPT to check methods section
**DATE REVIEW RETURNED**	09-Feb-2026

**GENERAL COMMENTS**	Thank you for this submission which is of interest to paediatricians, ED practitioners and researchers. A statistics review is required and I do not have the expertise to provide this. Please address comments below:<br>METHODS

	Study Design<br><br>Consider expanding “Retrospective cohort study” to specify that this is a population-based retrospective cohort using routinely collected linked healthcare data.<br><br>Data Used<br><br>The sentence “(420,051 attendances respectively)” is unclear — “respectively” appears unnecessary.<br><br>Please ensure consistent citation formatting (e.g. references 12 and 13).<br><br>Data Management<br><br>Specify whether analyses were conducted on attendance-level or individual-level data in each model to avoid ambiguity.<br><br>Definitions: Attendance Acuity<br><br>The low acuity definition is detailed and appropriate; however, a brief statement on validation or prior use of this definition would strengthen confidence.<br><br>Consider clarifying whether acuity was assessed per attendance or per individual.<br><br>DISCUSSION<br>While the discussion is clear it would benefit from greater interpretive depth and conceptual framing. Please expand on mechanisms related to findings, equity implications, and service design relevance. Consider how paediatric ED use differs conceptually from adult frequent attendance, especially in terms of caregiver decision-making.<br><br>Highlight how these findings could inform risk stratification or early intervention models

**REVIEWER NAME**	**Harron, Katie**
**REVIEWER AFFILIATION**	UCL Great Ormond Street Institute of Child Health
**REVIEWER CONFLICT OF INTEREST**	No Conflict of Interest
**AI DISCLOSURE**	No, I have not used AI
**DATE REVIEW RETURNED**	03-Mar-2026

**GENERAL COMMENTS**	These data were from almost 10 years ago. Are no more recent data available?<br><br>I understand from what is written, that you don’t have a complete year of follow up for those individuals aged <1 year. Is there any kind of sensitivity analysis you could do to address this?<br><br>Table 1 – please be consistent with your use of decimal places.<br><br>How did you deal with different values of the demographic variables (e.g. IMD) across multiple ED attendance records?

	What is driving the high level of missingness in the acuity variable?<br><br>Table 3 – please give age in order (and in Supplementary table 1).<br><br>How were missing data handled within the regression analyses?<br><br>The description in the main text is helpful for interpreting the quantile regression coefficients presented in Figures 1 and 2. It would also be helpful to give a concrete example here, i.e. of the increase in attendances compared to the reference category, within the older age group compared to the younger age group, for some quantile.<br><br>The final table in the appendix doesn't appear to have a title. What do the coefficients in this table represent?<br><br>Supplementary table 1 should make clear what the outcome is that is being modelled. The odds ratios / IRRs for the interaction terms in supplementary table 1 are fairly difficult to interpret without specifying the reference group. An alternative / additional way to present these effects would be to estimate the expected rates of the outcome within each of the strata. These could then be included in the table or plotted. Either way the coefficients for deprivation in the interaction terms should be ordered in the same way as the main effects (currently the main effects go from least to most deprived but this is the other way around for the interaction terms).<br><br>I'm not clear on what the logistic regression adds to the zero-truncated Poisson regression results. Could you justify why both approaches were used? I would suggest keeping one in as the main analysis and putting the other in the appendix.

VERSION 2 – AUTHOR RESPONSE

Reviewer: 1
METHODS
Study Design
Consider expanding "Retrospective cohort study" to specify that this is a population-based retrospective cohort using routinely collected linked healthcare data.

Thank you for this comment, the statement "Retrospective cohort study" has been replaced with the statement "A population-based retrospective cohort study using routinely collected linked healthcare data." in the abstract and in the methods.

Data Used
The sentence "(420,051 attendances respectively)" is unclear — "respectively" appears unnecessary.

Thank you for this, the redundant word "respectively" has now been omitted.

Please ensure consistent citation formatting (e.g. references 12 and 13).

Thank you for this comment the citations have been formatted consistently throughout.

Data Management
Specify whether analyses were conducted on attendance-level or individual-level data in each model to avoid ambiguity.

Thank you for this comment, the authors have now specified in the data preparation subsection that although the descriptive tables display both individual and attendance level output, the regression models were fit using individual-level information and the number of attendances made as the outcome. “Descriptive analyses were conducted on both attendance-level data and individual-level data, whereas regression models were fit using individual level data only.”

Definitions: Attendance Acuity

The low acuity definition is detailed and appropriate; however, a brief statement on validation or prior use of this definition would strengthen confidence.

Thank you for this comment. The low acuity variable was derived in a separate study and hence the definition described was not conceived by the authors of this manuscript. Validation of the acuity measurement was not possible in this study, and its limitation has been stated in the attendance acuity section.

Consider clarifying whether acuity was assessed per attendance or per individual.

Thank you for this comment, in the attendance acuity definition section we have stated the acuity is defined as an “attendance…meeting the following conditions” which clarifies this was an attendance-level measure.

DISCUSSION
While the discussion is clear it would benefit from greater interpretive depth and conceptual framing. Please expand on mechanisms related to findings, equity implications, and service design relevance. Consider how paediatric ED use differs conceptually from adult frequent attendance, especially in terms of caregiver decision-making.
Highlight how these findings could inform risk stratification or early intervention models

Thank you for this comment, the authors value your suggestion of highlighting how the findings could inform risk stratification and early intervention models. We have now added a paragraph in the implications for practice and further research to reflect this. “Unlike adult high frequency ED use, which is often driven by chronic disease or substance misuse, paediatric ED use is often mediated by caregiver health literacy and risk perception. The finding that younger age groups and those from deprived areas attend more frequently suggests a 'safety net' reliance where the ED compensates for limited access to primary care or caregiver anxiety regarding acute illness in infants. To move toward proactive care, these data should inform risk stratification models that identify 'rising risk' families triggering early intervention, potentially via health visitors or community paediatric hubs before a pattern of high-frequency use is established.”

Reviewer: 2
These data were from almost 10 years ago. Are no more recent data available?
Thank you for this comment, we are aware of this limitation and have included it in the limitation section. However, unfortunately there was no updated data available during the study analysis period. “As the data used in this study is over 8 years old, it may not reflect the recent behavioural and ED resource management shifts as a consequence of the covid-19 pandemic. However, this study can be used as a pre-covid comparison with more up-to-date analyses.”

I understand from what is written, that you don't have a complete year of follow up for those individuals aged <1 year. Is there any kind of sensitivity analysis you could do to address this?

This is correct, as some infants were born later in the study year, they will have less time to accrue ED attendances. However, since the rate ratios are already high for this demographic despite the underestimation, it strengthens the argument that the youngest children are the highest drivers of ED use.

Table 1 – please be consistent with your use of decimal places.

Thank you for this comment, the tables have now been amended to only contain 1 decimal place in the descriptive tables and 2 decimal places for the regression output tables.

How did you deal with different values of the demographic variables (e.g. IMD) across multiple ED attendance records?

To avoid multiple values for demographic data, patient-level information was taken at their first ED attendance date. This has now been included in the statistical analysis section: "The patient's sex, age, ethnicity and deprivation status were independent variables considered in all statistical analyses taken at their first ED attendance."

What is driving the high level of missingness in the acuity variable?

Thank you for this comment, unfortunately the missingness of the acuity variables is due to underlying missingness of variables such as investigation, treatment and disposal codes.

Table 3 – please give age in order (and in Supplementary table 1).

Thank you, this has now been amended.

How were missing data handled within the regression analyses?

Thank you for this comment and is an important question. The regression analysis was conducted using a complete-case sample and this has now been made clear in the methods section of the manuscript. "Regression analyses were conducted using the complete-case sample and thus individuals with missing data were omitted from this analysis."

The description in the main text is helpful for interpreting the quantile regression coefficients presented in Figures 1 and 2. It would also be helpful to give a concrete example here, i.e. of the increase in attendances compared to the reference category, within the older age group compared to the younger age group, for some quantile.

Due to the quantile regression for counts methodology, the corresponding output are not easily interpretable in the way that IRR and ORs are. This is due to the fact that the coefficients are related to a mathematical transformation of the ED attendance count variable (namely the natural log and random noise added). So the strength and direction of the coefficients are important but are reported alongside the more intuitive Poisson and logistic regression output for context.

The final table in the appendix doesn't appear to have a title. What do the coefficients in this table represent?

Thank you for this comment, a title has now been added "Output of the quantile regression for counts analysis across a range of quantiles".

Supplementary table 1 should make clear what the outcome is that is being modelled.

Thank you for this comment this has now been stated in the title of the table “Logistic and Zero Truncated (ZT) Poisson Regression with Interaction Term (Ethnicity & Deprivation) modelled using the binary high frequency attender (3+ attendances) and attendance count outcome variable respectively.”.

The odds ratios / IRRs for the interaction terms in supplementary table 1 are fairly difficult to interpret without specifying the reference group. An alternative / additional way to present these effects would be to estimate the expected rates of the outcome within each of the strata. These could then be included in the table or plotted. Either way the coefficients for deprivation in the interaction terms should be ordered in the same way as the main effects (currently the main effects go from least to most deprived but this is the other way around for the interaction terms).

Thank you, this is a useful comment. The interaction term reference category is individuals who are white and belonging to the least deprived IMD status. This has been added to the table. The ordering of the table output have also been amended as suggested.

I’m not clear on what the logistic regression adds to the zero-truncated Poisson regression results. Could you justify why both approaches were used? I would suggest keeping one in as the main analysis and putting the other in the appendix.

Thank you for this comment, the logistic regression analysis using a binary definition of high frequency use (3+ attendances) was included for two reasons. The first of which being, the vast majority of other predictors of frequent use published papers use a binary threshold and thus comparisons with our work is easier if presented in this way. Secondly the logistic regression in our analysis acts as a sensitivity analysis to the zero truncated model and thus verifies the findings.

VERSION 3 – REVIEW

**REVIEWER NAME**	**Harron, Katie**
**REVIEWER AFFILIATION**	UCL Great Ormond Street Institute of Child Health
**REVIEWER CONFLICT OF INTEREST**	No Conflict of Interest
**AI DISCLOSURE**	No, I have not used AI
**DATE REVIEW RETURNED**	27-May-2026

**GENERAL COMMENTS**	The authors have addressed my previous comments and I have nothing further to add.
